# High intra- and inter-observer reliability of the PJI-TNM classification in acute and chronic periprosthetic hip joint infection

**DOI:** 10.5194/jbji-11-323-2026

**Published:** 2026-06-04

**Authors:** Dominic Simon, Jonas Tumler, Lennart M. Schroeder, Florian Pachmann, Eduardo Suero, Boris M. Holzapfel, Gautier Beckers, Kathrin Pfahl

**Affiliations:** 1 Department of Orthopaedics and Trauma Surgery, Musculoskeletal University Center Munich (MUM), University Hospital, LMU Munich, Marchioninistr. 15, 81377 Munich, Germany

## Abstract

**Background**: Periprosthetic joint infections (PJIs) remain a significant complication following total hip arthroplasties (THAs), affecting patient outcomes and healthcare costs. The accurate classification of PJIs is crucial for guiding treatment decisions and improving patient management. The TNM classification system, commonly used in oncology, has previously been adapted for PJI to enhance our understanding of infection severity and is progressively used as PJI-TNM. **Purpose**: This study evaluates the applicability of the PJI-TNM classification in a cohort of 185 periprosthetic hip joint infections, including 84 acute and 101 chronic cases. **Methods**: In this retrospective study, we analyzed 84 cases of acute and 101 cases of chronic periprosthetic hip joint infections. Each case was independently classified according to the PJI-TNM framework by three observers. A second round of scoring was performed 90 d later to assess the intra-observer concordance. The objective was to evaluate the utility of this classification in distinguishing between acute and chronic infections, predicting outcomes, and informing treatment strategies. Furthermore, intra- and inter-observer reliability were evaluated. **Results**: For acute PJIs we found a high inter-observer and intra-observer reliability for both the first and second evaluation ( κ 0.78 and 0.89, respectively). In addition, the intra-observer analysis showed a very good correlation ( κ 0.86–0.96). For chronic PJIs the intra-observer reliability was κ of 0.85–0.96, whereas inter-observer reliability was lower with κ of 0.75–0.85. **Conclusion**: The PJI-TNM classification demonstrated strong intra-observer and substantial inter-observer reliability across both acute and chronic infections, supporting its use as a robust and reproducible framework for PJI classification.

## 1 Introduction

As the number of primary total hip arthroplasties (THAs) performed each year continues to grow, the overall volume of complications is expected to increase accordingly, with periprosthetic joint infection (PJI) remaining among the most serious (Tsikopoulos and Meroni, 2023). The numbers of performed total joint arthroplasty of the lower extremity are estimated to rise by up to 600 % until 2030, both in the United States and Europe (Kurtz et al., 2007; Leitner et al., 2018; Robertsson et al., 2010). The incidence of PJI after THA is estimated at 0.5 %–2 %, but the absolute burden is rising with arthroplasty volume (Aftab et al., 2025). PJI often requires complex revision surgery and prolonged antimicrobial therapy, and is associated with reduced functional outcomes and increased mortality. Furthermore, it is well known that it is associated with significant morbidity and mortality as well as a substantial economic burden on healthcare systems worldwide (Premkumar et al., 2021; Kamath et al., 2015; Kardi and Franceschi, 2020; Szymski et al., 2024).

Acute PJIs (early postoperative or acute hematogenous) more often involve higher-virulence organisms such as *Staphylococcus aureus* or β -hemolytic streptococci, whereas chronic infections more commonly feature biofilm-forming, low-virulence pathogens such as coagulase-negative staphylococci and *Cutibacterium acnes*. Biofilm formation on implant surfaces underpins diagnostic and therapeutic challenges by limiting antibiotic penetration and shielding bacteria from host defenses. In chronic infections, biofilm-embedded bacteria differ fundamentally from planktonic organisms typically seen in acute infections, contributing to diagnostic difficulty and reduced treatment efficacy (Bakalakos et al., 2024; Davidson et al., 2019; Rozis et al., 2021).

Overall, most cases involve Gram-positive cocci like *Staphylococcus aureus*, *Staphylococcus epidermidis*, and *Streptococcus pyogenes*, although Gram-negative bacteria such as *Pseudomonas aeruginosa* and fungi such as *Candida albicans* may also contribute (WHO, 2011).

The 2018 International Consensus Meeting (ICM) definition introduced a weighted, evidence-based scoring system for PJI diagnosis that has performed well on external validation and is now widely adopted in clinical research and practice (Parvizi et al., 2018).

Despite advances in diagnosis by combining clinical, laboratory, and imaging findings, diagnosing and treating PJI remains challenging due to its variable presentation and the influence of microbial and host factors.

Patient-related factors such as smoking, increased body mass index, and comorbidity burden are well-established risk factors influencing the development and severity of periprosthetic joint infections. Smoking has been associated with impaired immune response and wound healing (Bojan et al., 2020), while obesity contributes to systemic inflammation and altered host defense (Carender et al., 2024). Similarly, a higher comorbidity burden, commonly assessed using the Charlson comorbidity index (CCI), reflects reduced physiological reserve and increased susceptibility to infection, underscoring the relevance of these factors in stratifying infection severity in acute PJI (Kong et al., 2016).

A new TNM classification for PJI was proposed (Table 1), based on tissue and implant status, non-human cells with bacteria and fungi, and patient morbidities that could help to standardize care and improve research comparability (Rupp et al., 2021; Alt et al., 2020).

**Table 1:** TNM classification adapted from Alt et al. (2020).

TNM	Classification	Description
Tissue and implant status	0a 0b 1a 1b 2a 2b	Stable standard implant without important soft tissue defect Stable revision implant without important soft tissue defect Loosened standard implant without important soft tissue defect Loosened revision implant without important soft tissue defect Severe soft tissue defect with standard implant Severe soft tissue defect with revision implant
Non-human cells	0a 0b 1a 1b 2a 2b 2c	No mature biofilm formation (former: acute), directly postoperatively No mature biofilm formation (former: acute), late hematogenous Mature biofilm formation (former: chronic) without “difficult to treat bacteria” Mature biofilm formation (former: chronic) with culture-negative infection Mature biofilm formation (former: chronic) with “difficult to treat bacteria” Mature biofilm formation (former: chronic) with polymicrobial infection Mature biofilm formation (former: chronic) with fungi
Morbidities	0 1 2 3a 3b 3c	Not or only mildly compromised (Charlson comorbidity index: 0–1) Moderately compromised patient (Charlson comorbidity index: 2–3) Severely compromised patient (Charlson comorbidity index: 4–5) Patient refuses surgery Patient does not benefit from surgery Patient does not survive surgery
r		Recurrent infection of a previously infected implant “r” in front of the classification

The classification's structured approach may facilitate communication among healthcare providers and support future research into PJI outcomes. However, its prognostic value and direct clinical applicability remain to be established.

Therefore, the primary aim of this study was to determine the reproducibility of the PJI-TNM classification. In addition, we explored its relationship with patient-related factors and causative microorganisms in a descriptive manner, without implying prognostic validity.

We hypothesized that the TNM classification provides good intra- and inter-observer reliability in the classification of both acute and chronic PJIs.

## 2 Materials and methods

We conducted a retrospective university hospital single-center cohort study. A total of 309 THA patients were initially identified using ICD-10 code T84.5, which denotes infection and inflammatory reaction due to an internal joint prosthesis. Patients with incomplete medical records, those younger than 18 years, and those miscoded or lacking evidence of infection according to the Musculoskeletal Infection Society (MSIS) and EBJIS criteria were excluded. The final cohort consisted of 185 patients treated for PJI, defined according to MSIS and EBJIS criteria, between January 2013 and June 2024. Infection was confirmed with presence of a sinus tract or purulence surrounding the prosthetic component, synovial fluid analysis showing WBC count > 3000 cells µ L^−1^ or PMN > 80 %, intraoperative histology consistent with Krenn and Morawietz type II or III, or microbial growth detected in synovial fluid, in at least two periprosthetic tissue samples or in sonication fluid exceeding 50 CFU mL^−1^ (Vogely, 2021; Sousa et al., 2023; Parvizi et al., 2018; Ochsner et al., 2014). Both MSIS and EBJIS criteria were applied to reflect contemporary clinical practice and to ensure comprehensive and robust identification of PJI cases, as these definitions are widely accepted and considered complementary.

Patients who exhibited clinical symptoms of infection along with characteristic laboratory findings, such as elevated cell counts from diagnostic joint aspiration, but without microbiological growth, were classified as having culture-negative PJIs. Microbiological specimens were incubated for 14 d at the Institute of Microbiology prior to analysis, with this protocol remaining consistent throughout the study period.

All data were extracted from patients' electronic medical records. Observers were provided with identical information during both assessment rounds, including clinical data, imaging, microbiological findings, and operative reports. Furthermore, all observers were blinded to patient outcomes and treatment strategies at the time of classification to minimize potential bias. A 90 d interval between the two assessment rounds was selected to reduce potential recall bias while ensuring feasibility in the study design. Characteristics included age, sex, infection type (acute or chronic), body mass index (BMI), Charlson comorbidity index (CCI), American Society of Anesthesiologists (ASA) classification, time interval between implantation and onset of PJI, and treatment strategy (such as debridement, antibiotics, and implant retention (DAIR), double DAIR, one- or two-stage revision with spacer implantation, or the Girdlestone procedure). Additional variables included risk factors such as smoking and diabetes. The number and classification of microbiological findings were also recorded.

Endpoints: the TNM classification of all acute and chronic cases was independently performed by a final-year medical student with arthroplasty experience, a board-certified specialist, and a senior consultant at baseline (T0) and again at 90 d to assess intra-observer and inter-observer reliability (T90).

Patient demographics and clinical characteristics are presented in Table 2. The cohort included 84 acute and 101 chronic infections, comprising of 91 males and 94 females.

**Table 2:** Patient demographics.

	All ( n=185 )	Acute PJIs ( n=84 )	Chronic PJIs ( n=101 )
Mean age (years)	72.2 ± 12.1	73.6 ± 11.3	71.0 ± 12.6
Mean BMI (kg m^−2^)	27.1 ± 6.0	27.2 ± 6.5	27 ± 5.7
Sex			
Female	94 (50.8 %)	45 (53.6 %)	49 (48.5 %)
Male	91 (49.2 %)	39 (46.4 %)	52 (51.5 %)
ASA score	2.7 ± 0.5	2.8 ± 0.5	2.6 ± 0.5
CCI	2.1 ± 1.9	2.4 ± 2.0	1.9 ± 1.9
Time from index THA to event (months)	41 ± 75	0.9 ± 1.4	67 ± 97

BMI, body mass index; CCI, Charlson comorbidity index; ASA, American Society of Anesthesiologists; THA, total hip arthroplasty.

### Statistical analysis

Data were analyzed using Excel (v16.30; Microsoft Corporation, Redmond, WA, USA). Descriptive statistics were used to summarize demographic and clinical variables and were expressed as means, standard deviations, percentages, and confidence intervals, where applicable.

Intra- and inter-observer reliability of the PJI-TNM classification was assessed using Cohen's kappa coefficients. Correlation analyses were performed to examine associations between TNM classification and patient-related factors (age, sex, BMI, CCI, and smoking status), as well as with the distribution of causative bacteria in both acute and chronic PJIs.

Reliability was interpreted according to established thresholds: values < 0.40 were considered poor agreement, 0.40–0.59 fair agreement, 0.60–0.74 good agreement, and ≥ 0.75 excellent agreement (Cicchetti et al., 2006). These correlation analyses were exploratory in nature and intended to generate hypotheses, therefore results should be interpreted with caution. All statistical analyses were performed using GraphPad Prism 10.1.2 (GraphPad Software LLC, Boston, MA, USA) and R Statistical Software (version 2024.04.2 for macOS; R Core Team, Vienna, Austria).

## 3 Results

**Figure 1:**
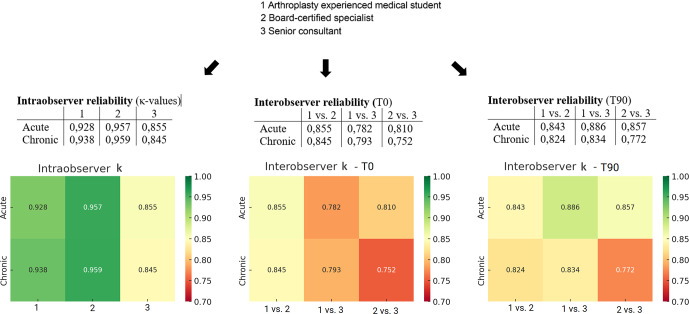
Intra-observer and inter-observer reliability ( κ values) at time point 0 (T0) and after 90 d (T90).

For acute PJI-TNM classifications, both intra-observer reliability (time points 0 and 90 d) and inter-observer reliability demonstrated excellent agreement with κ values consistently above 0.75 and reaching over 0.90 for several comparisons. For chronic PJI-TNM classifications, intra-observer reliability remained good ( κ=0.85 –0.96), whereas inter-observer reliability was slightly lower but still within the good range ( κ=0 .75–0.85 across comparisons), as presented in Fig. 1. This modest reduction in inter-observer agreement in chronic PJI likely reflects the greater clinical and morphological complexity of these cases, which may contribute to increased variability in classification.

**Figure 2:**
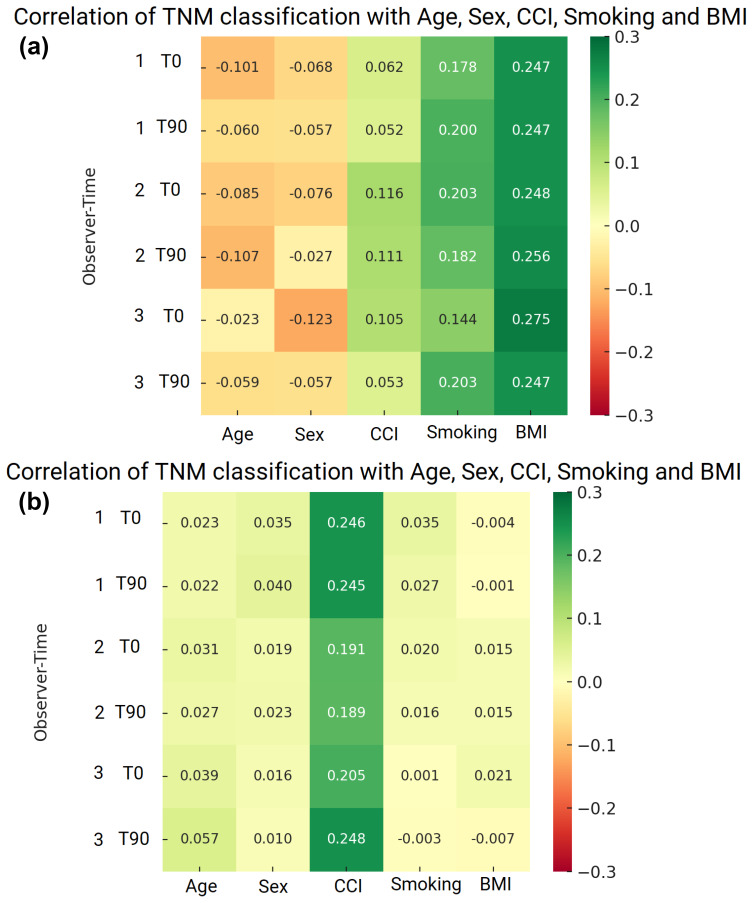
**(a)** Correlation of TNM classification with age, sex, CCI, smoking, and BMI in acute PJIs. **(b)** Correlation of TNM classification with age, sex, and Charlson comorbidity index in chronic PJIs.

In acute infections, there was a tendency toward higher TNM classifications in smokers ( r≈0 .14–0.20). BMI demonstrated the strongest positive correlation ( r≈0 .25–0.27), while the CCI showed a mild positive association. Age and male sex exhibited mild negative correlations with TNM classification, as presented in Fig. 2. Overall, these correlations were small to moderate in magnitude.

In chronic infections, the CCI showed the strongest positive correlation with TNM classification ( r≈0 .19–0.25). Age, sex, smoking status, and BMI demonstrated correlations close to zero. Overall, no patient-related factor showed a strong correlation with TNM classification in the chronic dataset.

In acute PJI, higher TNM classifications were most strongly associated with *Staphylococcus capitis*, *Streptococcus mitis*, *Pseudomonas aeruginosa*, and *Candida* species. Several organisms showed mild positive correlations, including *Acinetobacter baumannii*, *Enterococcus faecium*, and *Staphylococcus aureus*. Negative correlations were observed for *Staphylococcus epidermidis*, *Streptococcus dysgalactiae*, *Enterobacteriaceae*, anaerobes, and culture-negative infections. Overall, these associations were small to moderate and should be interpreted with caution.

In chronic PJI, higher TNM classifications demonstrated the strongest positive correlations with multi-bacterial infections, *Staphylococcus haemolyticus*, *Streptococcus dysgalactiae*, *Pseudomonas aeruginosa*, *Enterobacter* species, *Enterococci*, and *Escherichia coli*. These organisms were more frequently associated with advanced TNM staging; however, the strength of these correlations remained small to moderate.

**Figure 3:**
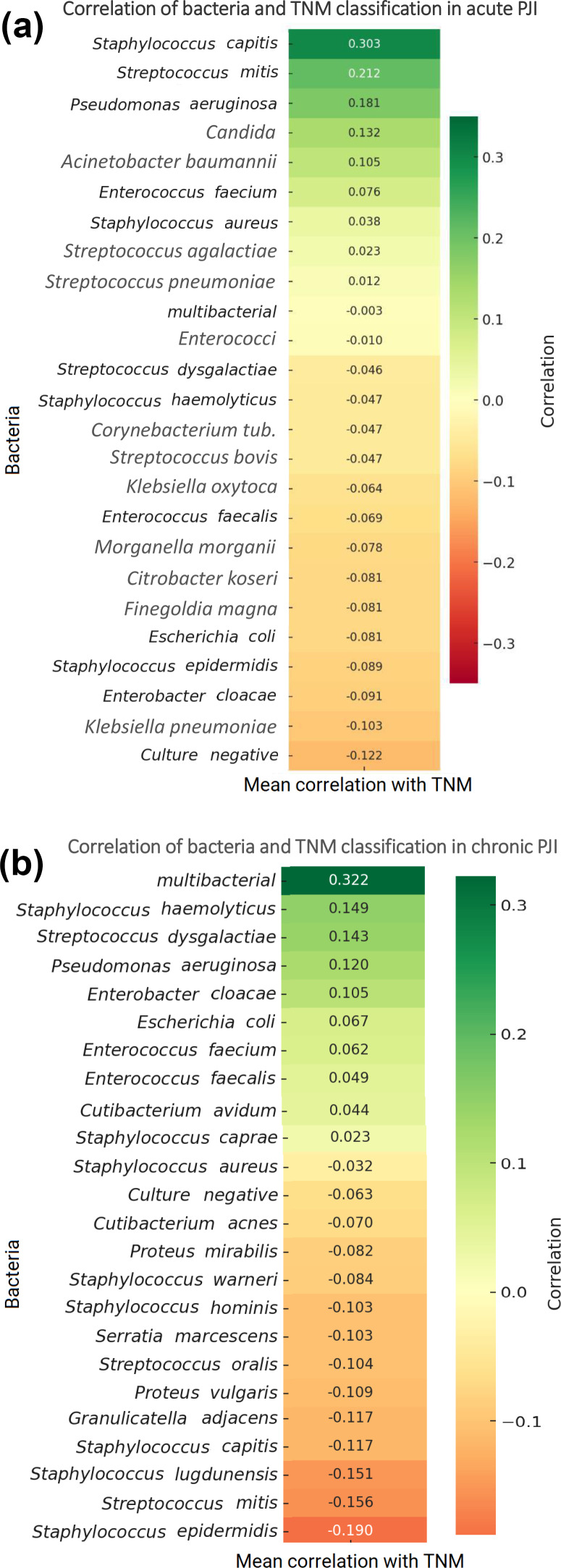
**(a)** Correlation of bacteria and TNM classification in acute PJIs. **(b)** Correlation of bacteria and TNM classification in chronic PJIs.

In contrast, *Staphylococcus epidermidis* and *Streptococcus mitis* showed low or weak correlations with TNM classification, indicating that these pathogens were more commonly found in cases with lower TNM stages. Overall, no single organism demonstrated a very strong association with TNM in chronic infections, but polymicrobial infections and certain Gram-negative bacteria showed the highest positive trends, as illustrated in Fig. 3.

## 4 Discussion

In this study, we assessed the reliability and applicability of the PJI-TNM classification in what is, to the best of our knowledge, the largest cohort of acute and chronic periprosthetic hip joint infections, demonstrating excellent intra-observer ( κ≈0 .86–0.96) and inter-observer ( κ≈0 .75–0.90) agreement, supporting the reproducibility of this classification system. The structured approach of the PJI-TNM classification may facilitate standardized assessment and communication among healthcare providers. Our findings indicate that the system can be applied consistently across clinicians with varying levels of experience, highlighting its practical feasibility in clinical and research settings.

These results align closely with the initial validation study by Baertl et al. (2024), which reported substantial reproducibility when applied by orthopedic specialists, with excellent intra-observer reliability for certain subdomains (e.g., reinfection category, 94.8 % accuracy) and moderate inter-observer reliability for others (e.g., implant condition).

Our findings extend this external validation to a larger cohort ( n=185 ) and reflect real-world application by clinicians with heterogeneous clinical experience (senior consultant, one board-certified specialist, and one arthroplasty-experienced medical student in their final year).

Moreover, our results are consistent with Lunz et al.'s (2023) work demonstrating significant correlations between preoperative PJI-TNM scores in chronic knee PJI managed with two-stage revision, underscoring the classification's prognostic value (Lunz et al., 2023; Alt et al., 2023).

In particular, our cohort showed similar patterns, with M-status (comorbidities) showing mild positive associations with TNM severity, supporting the idea that patient morbidity augments classification granularity and treatment prediction.

Beyond reliability, early outcome data suggest that PJI-TNM can serve as a prognostic tool. Fröschen et al. (2025) found that PJI-TNM classification predicted revision-free implant survival in hip and knee PJI populations, indicating utility in guiding long-term outcomes.

Although our study did not measure survival directly, our demonstration of classification consistency in both acute and chronic contexts supports the PJI-TNM system's use in future outcome research.

Furthermore, our study was not designed to assess prognostic performance or clinical outcomes, therefore no conclusions regarding treatment superiority, disease progression, or patient outcomes can be drawn from our data. Nevertheless, comparisons with alternative frameworks reveal further promise. Rocchi et al. (2025) reported that most available PJI classification systems exhibit limited inter-rater reliability when defining infection status, suggesting that PJI-TNM's structured, multi-dimensional format may offer better consistency and clinical applicability.

This adds weight to our conclusion that PJI-TNM offers improved reliability for clinicians managing PJIs, where the TNM framework could potentially support structured decision-making in future studies, while clearly emphasizing that this requires prospective outcome-based validation.

Notwithstanding these strengths, several limitations arise. Our retrospective single-center design may limit generalizability, and our raters, while diverse in experience, might still share institutional biases. Furthermore, while we assessed reliability comprehensively, we did not study outcome prediction such as reinfection rates. As Alt et al. (2023) suggest, future research should prospectively validate PJI-TNM against outcomes, such as reinfection, implant survival, or quality of life and across multi-center cohorts (Rupp et al., 2021).

The growing momentum toward prognostic validation (e.g., Kienzle et al., 2025, evaluating TNM with functional outcome metrics) underscores this need.

In summary, our study reinforces the reliability and clinical feasibility of the PJI-TNM classification in distinguishing acute versus chronic infections in THA. When used by clinicians spanning senior consultants to trainees, it demonstrated reproducible classification across time points. This foundation may support its future integration into treatment algorithms, multi-center research, and prognostic modeling. Moving forward, prospective validation, especially connecting TNM profiles with surgical outcomes and patient-reported measures, will be essential to confirm its ultimate clinical value.

## 5 Conclusion

The application of the PJI-TNM classification in categorizing acute and chronic periprosthetic hip joint infections proves valuable for clinical classification. High intra- and inter-observer agreement across different levels of clinical experience supports its robustness in routine clinical practice.

In acute PJIs, both intra- and inter-observer reliability were consistently high, highlighting the robustness of this framework in early infection scenarios. In chronic PJIs, intra-observer agreement remained strong, while inter-observer reliability was slightly lower, reflecting the greater diagnostic complexity of long-standing infections. Nevertheless, the overall consistency of the PJI-TNM system across time points and observers underscores its potential as a standardized tool to enhance clinical decision-making, improve comparability between studies, and support individualized treatment strategies. By providing a systematic framework for evaluating infection severity, the TNM classification could improve patient outcomes and optimize treatment strategies. Broader application and validation in prospective and multi-center settings will further clarify its utility in routine practice.

Future research should focus on validating this classification across larger, diverse populations and exploring its potential integration into clinical practice guidelines.

Additionally, exploring the relationship between TNM classification scores and specific treatment modalities could offer insights into optimizing management strategies for both acute and chronic infections.

## Data Availability

Data are available on request from the corresponding author.

## References

[bib1.bib1] Aftab MHS, Joseph T, Almeida R, Sikhauli N, Pietrzak JRT (2025). Periprosthetic Joint Infection: A Multifaceted Burden Undermining Arthroplasty Success. Orthop Rev (Pavia).

[bib1.bib2] Alt V, Rupp M, Langer M, Baumann F, Trampuz A (2020). Can the oncology classification system be used for prosthetic joint infection?: The PJI-TNM system. Bone Joint Res.

[bib1.bib3] Alt V, Walter N, Rupp M, Baertl S (2023). Comment on Lunz et al. Impact and Modification of the New PJI-TNM Classification for Periprosthetic Joint Infections. J. Clin. Med. 2023, 12, 1262. J Clin Med.

[bib1.bib4] Baertl S, Rupp M, Kerschbaum M, Morgenstern M, Baumann F, Pfeifer C, Worlicek M, Popp D, Amanatullah DF, Alt V (2024). The PJI-TNM classification for periprosthetic joint infections. Bone Joint Res.

[bib1.bib5] Bakalakos M, Ampadiotaki MM, Vlachos C, Sipsas N, Pneumaticos S, Vlamis J (2024). Molecular Mechanisms of Biofilm Formation on Orthopaedic Implants: Review of the Literature. Maedica (Bucur).

[bib1.bib6] Bojan B, Perni S, Prokopovich P (2020). Systematic Review and Meta-Analysis of Tobacco Use as a Risk Factor for Prosthetic Joint Infection After Total Hip Replacement. Arthroplast Today.

[bib1.bib7] Carender CN, Fruth KM, Lewallen DG, Berry DJ, Abdel MP, Bedard NA (2024). Obesity and Primary Total Hip Arthroplasty: The Absolute versus Relative Risk of Periprosthetic Joint Infection at 15 Years. J Arthroplasty.

[bib1.bib8] Cicchetti D, Bronen R, Spencer S, Haut S, Berg A, Oliver P, Tyrer P (2006). Rating scales, scales of measurement, issues of reliability: resolving some critical issues for clinicians and researchers. J Nerv Ment Dis.

[bib1.bib9] Davidson DJ, Spratt D, Liddle AD (2019). Implant materials and prosthetic joint infection: the battle with the biofilm. EFORT Open Reviews.

[bib1.bib10] Fröschen FS, Greber L, Molitor E, Hischebeth GTR, Franz A, Randau TM (2025). The PJI-TNM Classification as Predictor for Revision-Free Implant Survival Rates in Patients with Periprosthetic Joint Infection of the Hip or Knee Joint. Infect Dis Rep.

[bib1.bib11] Kamath AF, Ong KL, Lau E, Chan V, Vail TP, Rubash HE, Berry D, Bozic K (2015). Quantifying the burden of revision total joint arthroplasty for periprosthetic infection. J Arthroplasty.

[bib1.bib12] Kardi EM, Franceschi F (2020). Prosthetic joint infection. A relevant public health issue. J Infect Public Health.

[bib1.bib13] Kienzle A, Walter S, Köhli P, Gwinner C, Hardt S, Müller M, Perka C, Donner S (2025). Assessing the TNM Classification for Periprosthetic Joint Infections of the Knee: Predictive Validity for Functional and Subjective Outcomes. J Pers Med.

[bib1.bib14] Kong L, Cao J, Zhang Y, Ding W, Shen Y (2016). Risk factors for periprosthetic joint infection following primary total hip or knee arthroplasty: a meta-analysis. Int Wound J.

[bib1.bib15] Kurtz S, Ong K, Lau E (2007). Projections of primary and revision hip and knee arthroplasty in the United States from 2005 to 2030. J Bone Joint Surg Am.

[bib1.bib16] Leitner L, Türk S, Heidinger M (2018). Trends and Economic Impact of Hip and Knee Arthroplasty in Central Europe: Findings from the Austrian National Database. Sci Rep.

[bib1.bib17] Lunz A, Lehner B, Voss MN, Knappe K, Jaeger S, Innmann MM, Renkawitz T, Omlor GW (2023). Impact and Modification of the New PJI-TNM Classification for Periprosthetic Joint Infections. J Clin Med.

[bib1.bib18] Ochsner PE, Borens O, Bodler PM (2014). Infections of the Musculoskeletal System: Basic Principles, Prevention, Diagnosis and Treatment.

[bib1.bib19] Parvizi J, Tan TL, Goswami K, Higuera C, Della Valle C, Chen AF, Shohat N (2018). The 2018 Definition of Periprosthetic Hip and Knee Infection: An Evidence-Based and Validated Criteria. J Arthroplasty.

[bib1.bib20] Premkumar A, Kolin DA, Farley KX, Wilson JM, McLawhorn AS, Cross MB (2021). Projected economic burden of periprosthetic joint infection of the hip and knee in the United States. J Arthroplasty.

[bib1.bib21] Robertsson O, Bizjajeva S, Fenstad AM (2010). Knee arthroplasty in Denmark, Norway and Sweden. A pilot study from the Nordic Arthroplasty Register Association. Acta Orthop.

[bib1.bib22] Rocchi C, Di Maio M, Bulgarelli A, Chiappetta K, La Camera F, Grappiolo G, Loppini M (2025). Agreement Analysis Among Hip and Knee Periprosthetic Joint Infections Classifications. Diagnostics (Basel).

[bib1.bib23] Rozis M, Evangelopoulos DS, Pneumaticos SG (2021). Orthopedic Implant-Related Biofilm Pathophysiology: A Review of the Literature. Cureus.

[bib1.bib24] Rupp M, Kerschbaum M, Freigang V, Bartl S, Baumann F, Trampuz A, Alt V (2021). PJI-TNM as new classification system for periprosthetic joint infections: An evaluation of 20 cases. Orthopade.

[bib1.bib25] Sousa R, Ribau A, Alfaro P, Burch MA, Ploegmakers J, McNally M, Clauss M, Wouthuyzen-Bakker M, Soriano A (2023). The European Bone and Joint Infection Society definition of periprosthetic joint infection is meaningful in clinical practice: a multicentric validation study with comparison with previous definitions. Acta Orthop.

[bib1.bib26] Szymski D, Walter N, Hierl K, Rupp M, Alt V (2024). Direct Hospital Costs per Case of Periprosthetic Hip and Knee Joint Infections in Europe – A Systematic Review. J Arthroplasty.

[bib1.bib27] Tsikopoulos K, Meroni G (2023). Periprosthetic Joint Infection Diagnosis: A Narrative Review. Antibiotics.

[bib1.bib28] Vogely C (2021). The EBJIS definition of periprosthetic joint infection. Bone Joint J.

[bib1.bib29] World Health Organization (2011). Report on the Burden of Endemic Health Care-Associated Infection Worldwide: Clean Care Is Safer Care.

